# Studying the Factors of Human Carotid Atherosclerotic Plaque Rupture, by Calculating Stress/Strain in the Plaque, Based on CEUS Images: A Numerical Study

**DOI:** 10.3389/fninf.2020.596340

**Published:** 2020-11-24

**Authors:** Zhenzhou Li, Yongfeng Wang, Xinyin Wu, Xin Liu, Shanshan Huang, Yi He, Shuyu Liu, Lijie Ren

**Affiliations:** ^1^Department of Ultrasound, Shenzhen Second People's Hospital, The First Affiliated Hospital of Shenzhen University Health Science Center, Shenzhen, China; ^2^School of Biomedical Engineering, Sun Yat-sen University, Guangzhou, China; ^3^Guangdong Academy Research on Virtual Reality (VR) Industry, Foshan University, Foshan, China; ^4^Department of Neurosurgery, Shenzhen Second People's Hospital, The First Affiliated Hospital of Shenzhen University Health Science Center, Shenzhen, China; ^5^School of Pharmacy, Sun Yat-sen University, Guangzhou, China; ^6^Department of Neurology, Shenzhen Second People's Hospital, The First Affiliated Hospital of Shenzhen University Health Science Center, Shenzhen, China

**Keywords:** carotid atherosclerotic plaque, vulnerable plaque, cardiovascular diseases, neovascularization, intraplaque hemorrhage, contrast-enhanced ultrasound, computational simulation

## Abstract

Carotid plaque neovascularization is one of the major factors for the classification of vulnerable plaque, but the axial force effects of the pulsatile blood flow on the plaque with neovessel and intraplaque hemorrhage was unclear. Together with the severity of stenosis, the fibrous cap thickness, large lipid core, and the neovascularization followed by intraplaque hemorrhage (IPH) have been regarded as high-risk features of plaque rupture. In this work, the effects of these factors were evaluated on the progression and rupture of the carotid atherosclerotic plaques. Five geometries of carotid artery plaque were developed based on contrast-enhanced ultrasound (CEUS) images, which contain two types of neovessel and IPH, and geometry without neovessel and IPH. A one-way fluid-structure interaction model was applied to compute the maximum principal stress and strain in the plaque. For that hyper-elastic and non-linear material, Yeoh 3rd Order strain energy density function was used for components of the plaque. The simulation results indicated that the maximum principal stress of plaque in the carotid artery was higher when the degree of the luminal stenosis increased and the thickness of the fibrous cap decreased. The neovessels within the plaque could introduce a 2.5% increments of deformation in the plaque under the pulsatile blood flow pressure. The IPH also contributed to the increased risk of plaque rupture that a gain of stress was 8.983, 14.526, and 34.47 kPa for the plaque with 50, 65, and 75%, respectively, when comparing stress in the plaque with IPH distributed at the middle to the shoulder of the plaque. In conclusion, neovascularization in the plaque could reduce the stability of the plaque by increasing the stress within the plaque. Also, the risk of plaque rupture increased when large luminal stenosis, thin fibrous cap, and IPH were observed.

## 1. Introduction

Carotid atherosclerotic plaque is one of the most common causes of stroke (Rossi et al., 2002; Murata et al., 2020), based on the fibrous cap, lipid core, and endothelial denudation with superficial platelet aggregation being closely associated with the incident (Sadat et al., 2009). Thin fibrous cap and large lipid core are the major factors of the vulnerable plaque and high risk of the plaque rupture (Falk et al., 1995; Naghavi et al., 2003; Finn et al., 2010), as rupture is commonly found in the plaque with the thickness of the fibrous cap <0.065 mm and the volume of the lipid core accounting for 40% of the total plaque volume (Kolodgie et al., 2001). Additionally, intraplaque hemorrhage (IPH) is also considered as a risk factor for the occurrence of future cardiovascular events based on previous investigations (Takaya et al., 2006; Crombag et al., 2019; Saba et al., 2019a).

Neovascularization in the carotid atherosclerotic plaque is the maker of a high risk of the vulnerable plaque (Moreno et al., 2004; Dunmore et al., 2006; Huang et al., 2008; Van der Veken et al., 2016; Demeure et al., 2017). As early as 1999, McCarthy et al. (1999) made a histological examination of 28 patients with carotid arteries, and found that neovessels were commonly found in the fibrous cap of atherosclerotic plaque and most of them existed in the medial and lateral corners of the plaque, but rarely at the bottom. Neovessels can become leakage sites of blood vessels through the aggregation of the inflammatory cells that increase the vulnerability of the plaque, and even lead to IPH (Dunmore et al., 2006; Moreno et al., 2006). Contrast-enhanced ultrasound (CEUS) imaging has been applied to assess the neovascularization in carotid atherosclerosis plaques, for additional evaluation of the vulnerable plaque during the screening procedure for stroke in clinical practice (Feinstein, 2006; Vicenzini et al., 2007; Schinkel et al., 2020; Zamani et al., 2020). Xiong et al. (2009) reported that symptomatic patients had more intense contrast agent within the plaque than asymptomatic patients. Denmark and Marcin (2011) reported that the increased density of the neovascularization was associated with the increasing vulnerability degree of the carotid atherosclerosis plaque.

Hemodynamic blood flow plays an important role in determining plaque progression and ruptures (Radwa and Eldosoky, 2020). Computational fluid dynamics (CFD) analysis of the carotid atherosclerotic plaque has been used to assess the distribution of the superficial dynamic stress and strain of the plaque (Tang et al., 2003; Woorak et al., 2017). Li Z. et al. (2006) have performed stress analysis of the carotid plaque with various fibrous cap thickness in the geometry derived from *in vivo* magnetic resonance imaging (MRI) by using CFD, showing that the morphology and component of the plaque were significantly related to the stability of the plaque (Li Z. Y. et al., 2006). Impacts of the plaque eccentricity and hemodynamics environment were also related to the stress distribution in the plaque and rupture site, according to the study of Tang et al. (2003). These studies have taken into account the geometric and structural factors, including luminal stenotic degree, thin fibrous cap, large lipid core, and so on. Previous studies reported that the density of neovessels was positively associated with destabilization of the plaque based on the 2D transverse simulation (Lu et al., 2015; Guo et al., 2018, 2019). Teng et al. (2012) established a 2D transverse CFD model based on carotid artery endarterectomy slices, and four carotid plaque samples were used to analyze the local mechanical environment of the neovessels. Their results indicate that the local mechanical environment of the neovessel could lead to the occurrence of the IPH. Nevertheless, in their investigation, the effect of the carotid artery blood flow was not included, and the impact of axial force from pulsatile blood flow on the stability of the plaque was ignored.

In this study, numerical simulation with one-way fluid–structure interaction was conducted on the theoretical geometries of carotid atherosclerotic plaque with neovascularization and IPH. The stress and strain distribution in the plaque were evaluated regarding various plaque formations. The 2-dimensional axial plaque geometries were established based on the findings from the CEUS imaging. The thickness of the fibrous cap and the degrees of lumen stenosis were taken into account to study the impacts of neovessels and IPH on the destabilization of the carotid atherosclerotic plaque.

## 2. Methods

### 2.1. Geometries

**Figure 1:**
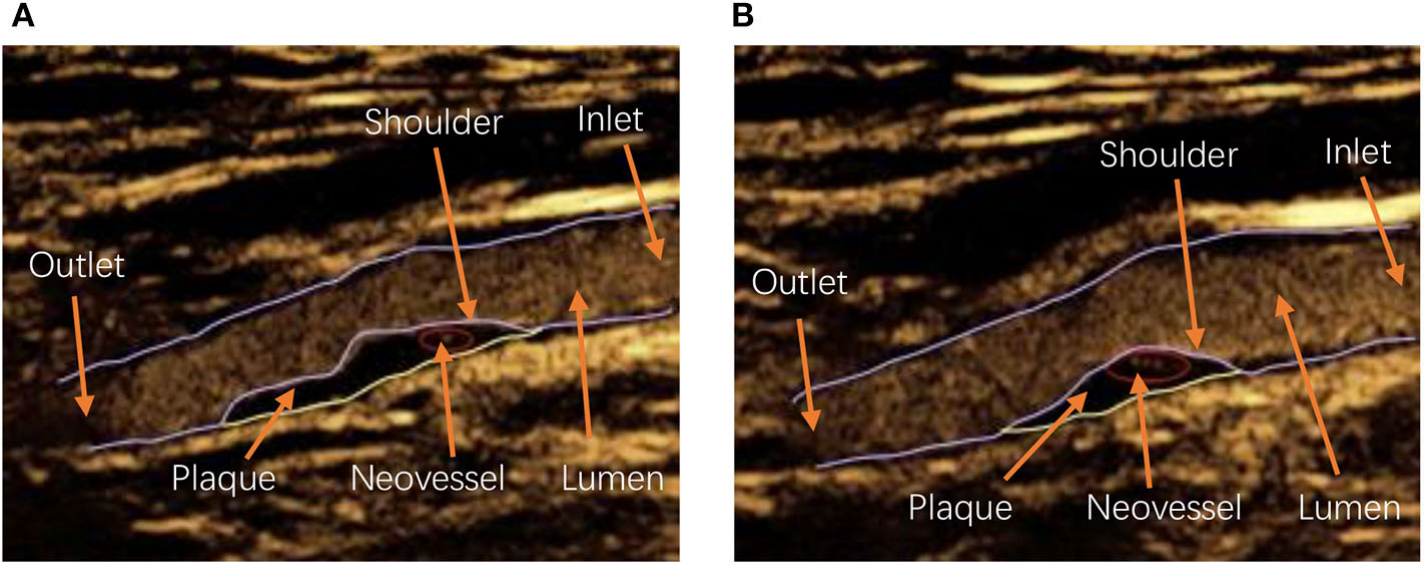
The contrast-enhanced ultrasound (CEUS) image with carotid artery lumen, atherosclerotic plaque, and neovessel: **(A)** is the CEUS image with one neovessel, and **(B)** is the CEUS image with three neovessels. Inlet and outlet: The carotid artery blood flow inlet and outlet, respectively; Shoulder: the area at the periphery of the plaque adjacent to the normal intima beneath the fibrous cap of the plaque (Pasterkamp et al., 1998).

**Figure 2:**
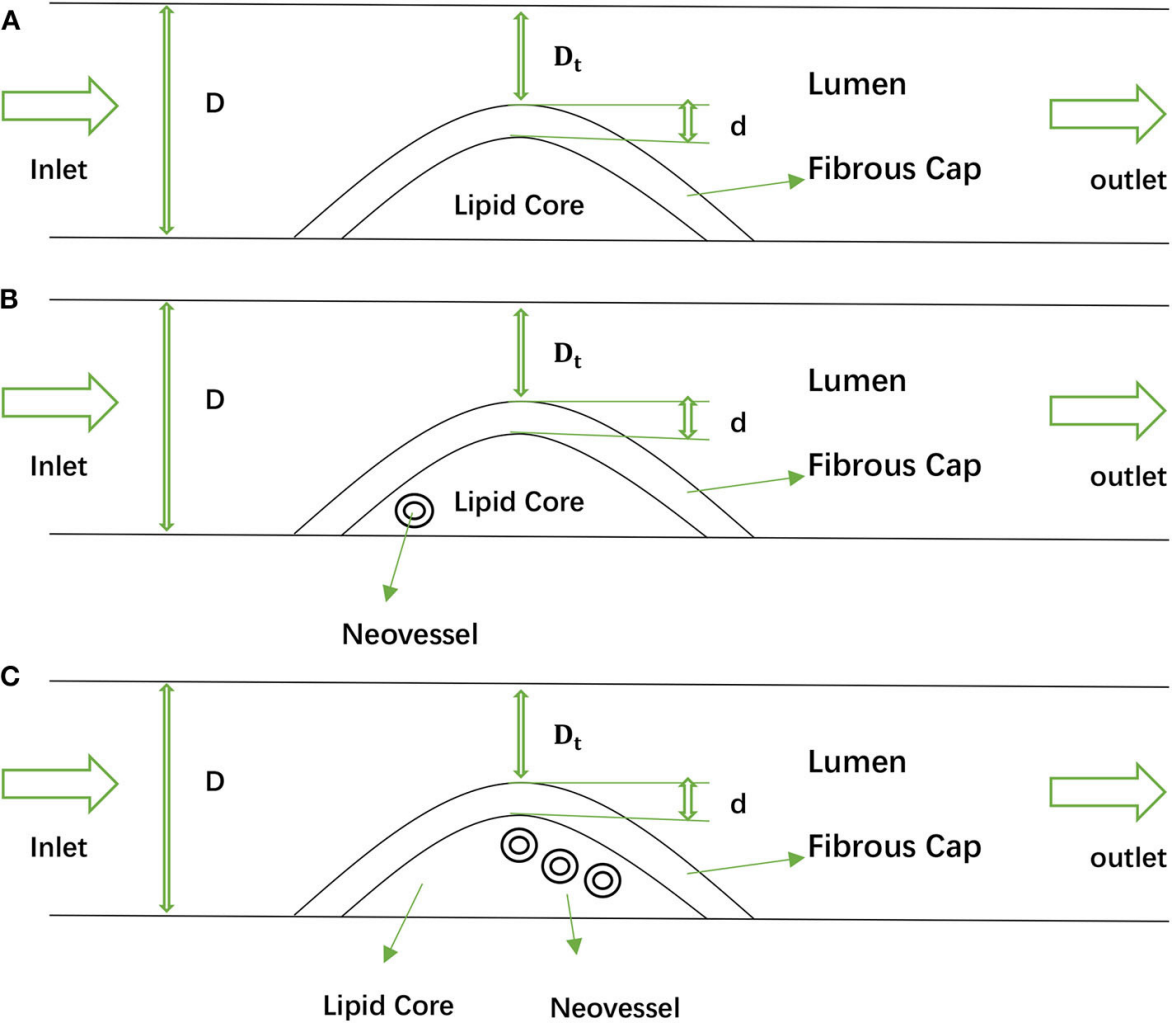
The 2D geometries of the idealized carotid artery with plaque and neovessel: **(A)** is the geometry with no neovessel, **(B)** is the geometry with one neovessel, and **(C)** is the geometry with three neovessels.

In this study, a one-way fluid–structure interaction model was performed to explore how neovessel and IPH affected carotid atherosclerotic plaque. Considering the structures of the plaque and neovessel occurring variation in different patients and even in the same one [see Figure 1: (a) is the carotid plaque with one neovessel, (b) is the one with three neovessels], 2D geometries based on CEUS images were taken into account in this study [see Figure 2: (a) is geometry with no neovessel, (b) is geometry with one neovessel, and (c) is one with three neovessels]. The structures of these geometries included carotid lumen, fibrous cap, lipid core, and different neovessels (Naghavi et al., 2003). For analysis, one inlet and outlet were necessary. The shapes of the fibrous cap and lipid core were designed by using a sinusoidal function (Li Z. Y. et al., 2006):

(1) $$\begin{array}{r} y_{1}=\frac{D-D_{t}}{2}\cdot cos\left(x\right) \end{array}$$

(2) $$\begin{array}{r} y_{2}=\frac{D-D_{t}}{2}\cdot \left(1+cos\left(x\right)\right)-d \end{array}$$

(3) $$\begin{array}{r} S=\frac{D}{D_{t}}\cdot 100\% \end{array}$$

where (*i*) D and *D*_*t*_ represent the diameter of the carotid lumen and the distance between the carotid artery wall and the top of the plaque, and (*ii*) S represents the degree of the carotid artery stenosis. The thickness of the fibrous cap, represented by d, plays a critical influence factor in the vulnerable plaque development (Cicha et al., 2011); different d values were used (d = 0.2, 0.5, and 0.0065 mm), which is critical to plaque vulnerability. In this paper, we set the stenosis degree S at 50, 65, and 75% (Avci et al., 2016). Moreover, the carotid artery lumen diameter of the geometry was assumed to be 10 mm. The lengths of the carotid artery and the plaque are set to 100 and 20 mm, respectively. The distance between the inlet boundary and the plaque structure was set to be 32 mm, and the outlet boundary was set to be 48 mm away from the plaque structure (Belzacq et al., 2012). Additionally, the diameter of the neovessel with 0.1 mm thickness was set at 0.4 mm. For the IPH, it was assumed to be a half-moon shape (Teng et al., 2014, 2015). Two types of IPH were used in this paper with the position of one IPH set in the shoulder of plaque, and another one in the middle of the plaque (see Supplementary Figure 1).

### 2.2. Computational Models, Materials, and Boundary Conditions

In this study, the components of the plaque were assumed to be hyper-elastic and non-linear because the ideal human tissue is hyper-elastic. The Yeoh 3rd Order strain energy density function was used to describe the material of the plaque in the carotid artery (Teng et al., 2015).

(4) $$\begin{array}{r} W=\overset{3}{\underset{i}{\sum }}C_{i}{\left(I_{i}-3\right)}^{i}+k\left(J-1\right),i=1,2,3 \end{array}$$

(5) $$\begin{array}{r} I_{1}={\lambda_{1}}^{2}+{\lambda_{2}}^{2}+{\lambda_{3}}^{2} \end{array}$$

where (i) *I*_*i*_ represents the first invariant of the unimodular component of the Cauchy–Green deformation tensor, (ii) *C*_*i*_ is the material constants, (iii) λ_*i*_ is the principal stretch, (iv) J = def (F) and F is the deformation gradient, (v) k is the Lagrangian multiplier for incompressibility, and (vi) J is equal to 0 for material incompressibility. The parameter values were chose to describe the material models (see Table 1): (i) fibrous cap: C1 = 53.724 kPa, C2 = 2201.011 kPa, C3 = 42.551 kpa, (ii) lipid core: C1 = 18.548 kPa, C2 = 207.371 kPa, C3 = 422.652 kPa, (iii) IPH: C1 = 11.225 kPa, C2 = 69,214 kPa, and C3 = 781.546 kPa, (iv) neovessel: C1 = 5.656 kPa, C2 = 1816.773 kPa, C3 = 162.037 kPa. The density and viscosity are the fundamental blood parameters. Generally, the range of blood density is 1,030–1.070 kg/m^3^. In our study, the constant density 1.050 kg/m^3^ was used, and the value of the blood viscosity is 3.400 Ns/m^2^ (Marshall et al., 2004). The blood was assumed to be an incompressible, laminar, and Newtonian fluid.

**Table 1:** The parameter values of the Yeoh model used in this paper.

	**C1 (kPa)**	**C2 (kPa)**	**C3 (kPa)**
Fibrous cap	53.724	2201.011	42.551
Lipid core	18.548	207.371	422.652
Neovessel	5.656	1816.773	162.037
IPH	11.225	69.214	781.546

**Figure 3:**
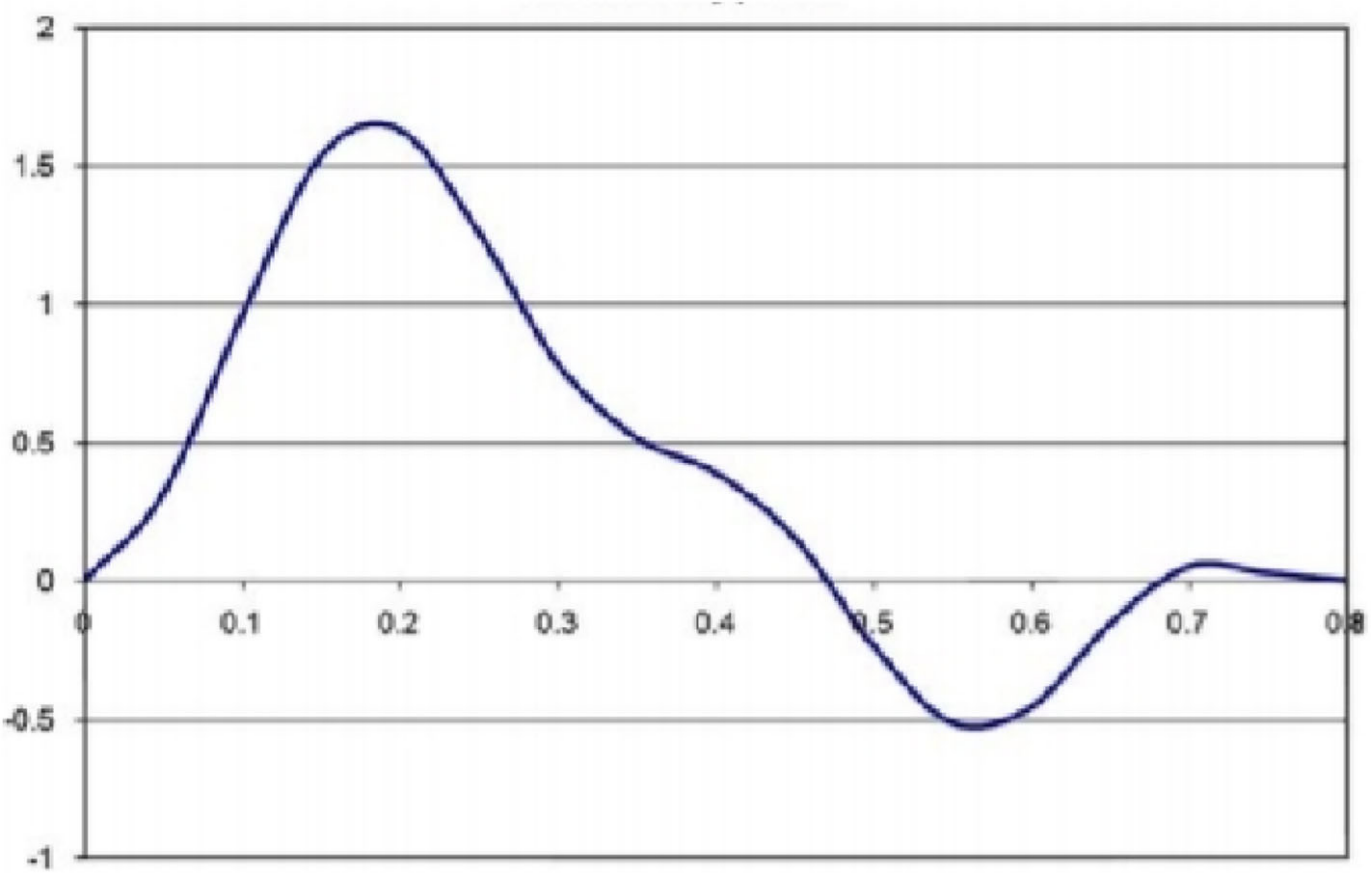
The velocity condition specified at the inlet boundary.

Additionally, the inlet boundary condition was transient with time (see Figure 3). This trend of the velocity changed over time, and it was close to the experimental curves (Viedma et al., 1997). After one cardiac cycle, the blood flow of the carotid outlet tended to be stable, and the relative pressure of the outlet was set as 0 Pa. For simulating the blood flow, the conditions of no-slip and non-deformation were considered at the carotid artery wall. The 2D computational models were solved by using ANSYS2019 R1. The one-way fluid–structure interaction was operated. The pressure of the fluid was loaded in the whole plaque, and an approximate blood pressure of 0.00133 MPa in the microvessel was exerted on the neovessel and IPH.

## 3. Results

**Figure 4:**
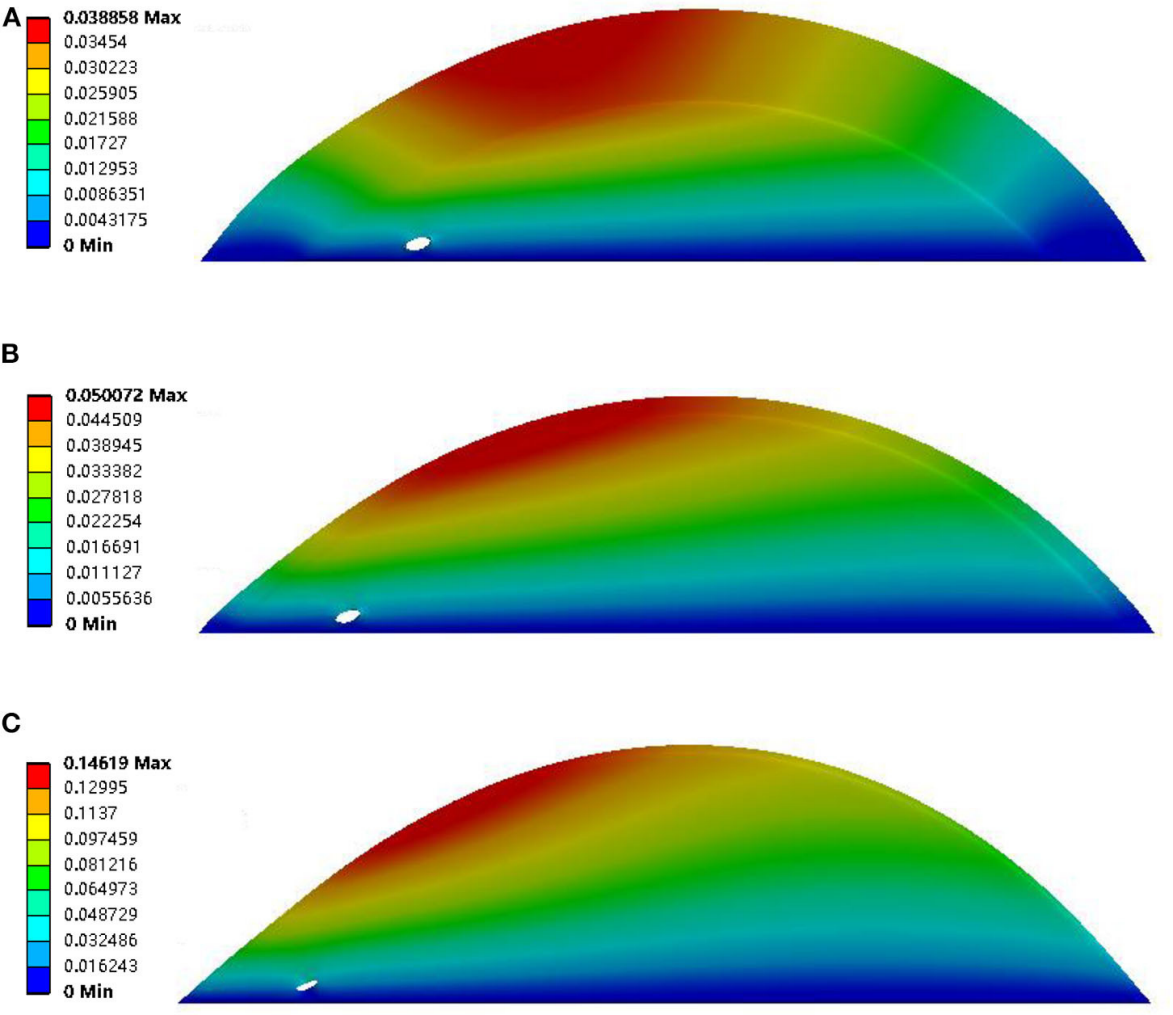
Deformation results of plaque with 50% luminal stenosis, one neovessel, and different thickness of the fibrous cap. The fibrous cap thicknesses of subfigures **(A–C)** were 2, 0.5, and 0.0065 mm, respectively.

**Figure 5:**
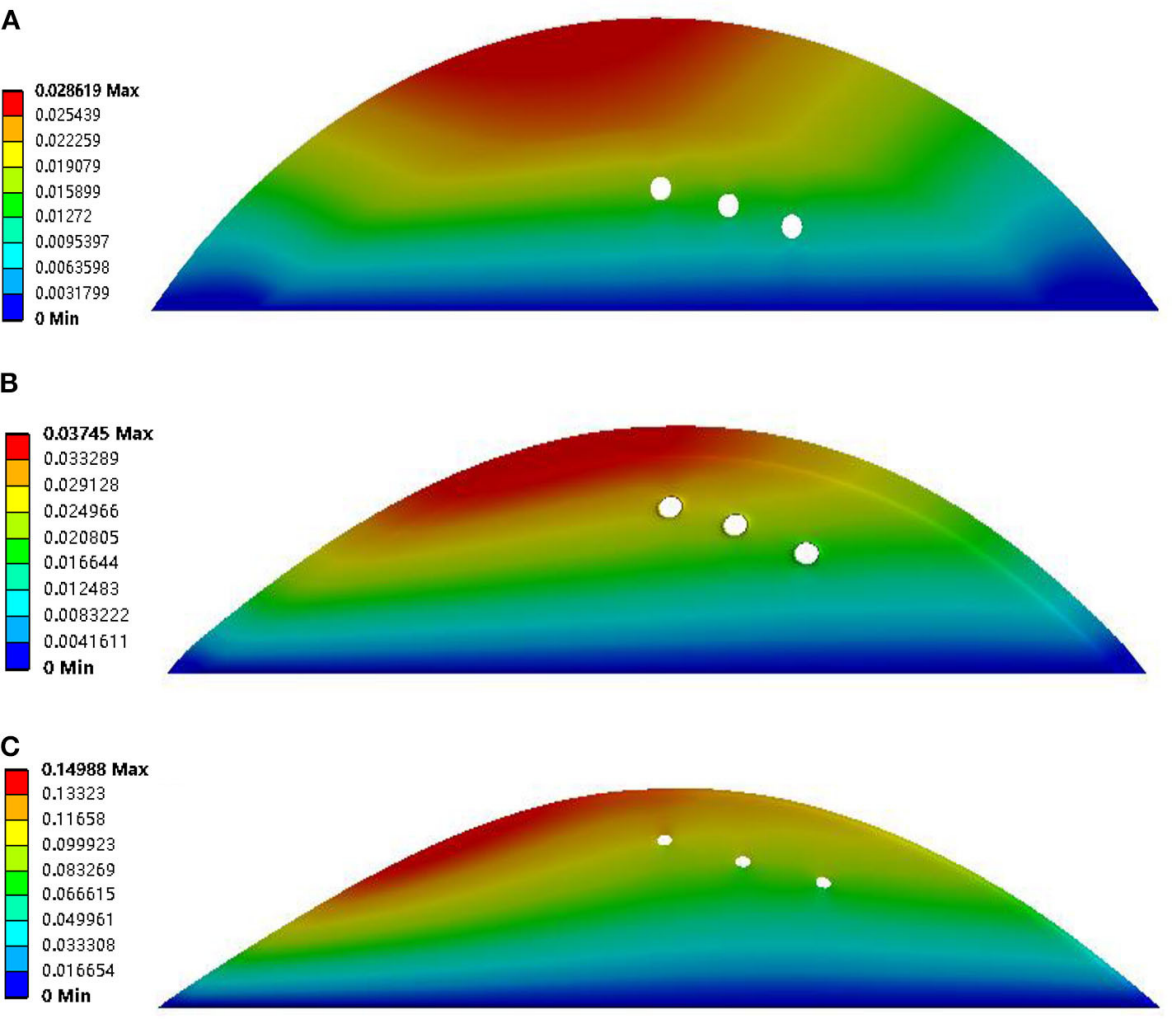
Deformation results of plaque with 50% luminal stenosis, three neovessel, and different thickness of fibrous cap. The fibrous cap thicknesses of subfigures **(A–C)** were 2, 0.5, and 0.0065, respectively.

Quantification parameters were evaluated in the present study. These parameters included total deformation, maximum principal stress, and maximum principal strain. The detail of the parameters was summarized in Table 2. The deformation of the plaques was negatively related to the thickness of the fibrous cap. The largest deformation was found in the geometry with a thin fibrous cap (thickness was 0.0065 mm), and it was 0.14619 (see Figure 4C). Also, the deformation of the neovessel was in line with the observation in the CEUS images that the shape of neovessel changes along with the pulsatile blood flow, which suggested that the stress on the plaque could further induce the stress on the neovessel. Moreover, the neovessel mainly consisted of endothelial cells without smooth muscle cells, which makes the neovessel vulnerable to the stress. Additionally, the deformation of the plaque with three neovessels (50% luminal stenosis, different thickness of fibrous cap) showed that the diameter of the neovessels was further suppressed when the fibrous cap was thin (see Figure 5). The largest deformation occurred in the plaque with 0.0065 mm fibrous cap thickness and the value of deformation is 0.14988 (see Figure 5C). It can be found that there was a relatively large deformation difference between the plaque with one neovessel and with three neovessels. The reason may be that the neovessel located closed to the plaque shoulder suffered from a larger pressure than the other three neovessels located in the plaque middle-lower stream.

**Table 2:** Comparison of maximum principal strain [MP strain (mm\mm)] and stress [MP stress (kPa)] of plaque during one cardiac cycle among no neovessel, one neovessel, three neovessels, and intraplaque hemorrhage (IPH).

**Degree of plaque stenosis (%)**	**Thickness of fibrous cap (mm)**	**Plaque geometry**	**MP strain (mm/mm)**	**MP stress (kPa)**
50	2	No neovessel and IPH	0.00116	11.6
		One neovessel	0.020946	11.625
		Three neovessels	0.019249	11.565
		IPH in plaque shoulder	0.016795	11.54
		IPH in plaque middle	0.017509	11.826
	0.5	No neovessel and IPH	0.015697	21.276
		One neovessel	0.021508	21.391
		Three neovessels	0.018977	21.398
		IPH in plaque shoulder	0.048537	74.321
		IPH in plaque middle	0.078302	71.435
	0.0065	No neovessel and IPH	0.058363	276.99
		One neovessel	0.06425	272.81
		Three neovessels	0.059618	269.89
		IPH in plaque shoulder	0.052972	296.63
		IPH in plaque middle	0.058464	272.28
65	2	No neovessel and IPH	0.019593	36.25
		One neovessel	0.021154	36.389
		Three neovessels	0.020147	36.178
		IPH in plaque shoulder	0.019791	36.673
		IPH in plaque middle	0.032817	37.965
	0.5	No neovessel and IPH	0.016165	54.49
		One neovessel	0.02203	54.871
		Three neovessels	0.018698	54.393
		IPH in plaque shoulder	0.015754	56.731
		IPH in plaque middle	0.015987	54.19
	0.0065	No neovessel and IPH	0.066529	563.67
		One neovessel	0.066929	575.43
		Three neovessels	0.066642	569.73
		IPH in plaque shoulder	0.068731	608.05
		IPH in plaque middle	0.06663	565.72
75	2	No neovessel and IPH	0.054892	225.72
		One neovessel	0.057081	226.87
		Three neovessels	0.056651	225.66
		IPH in plaque shoulder	0.055234	231.15
		IPH in plaque middle	0.053922	226.95
	0.5	No neovessel and IPH	0.050985	336.36
		One neovessel	0.051242	335.89
		Three neovessels	0.051038	333.86
		IPH in plaque shoulder	0.052625	350.08
		IPH in plaque middle	0.051274	336.18
	0.0065	No neovessel and IPH	0.10114	1242.6
		One neovessel	0.10223	1270.3
		Three neovessels	0.10125	1250.3
		IPH in plaque shoulder	0.10643	1358.8
		IPH in plaque middle	0.10206	1261.5

**Figure 6:**
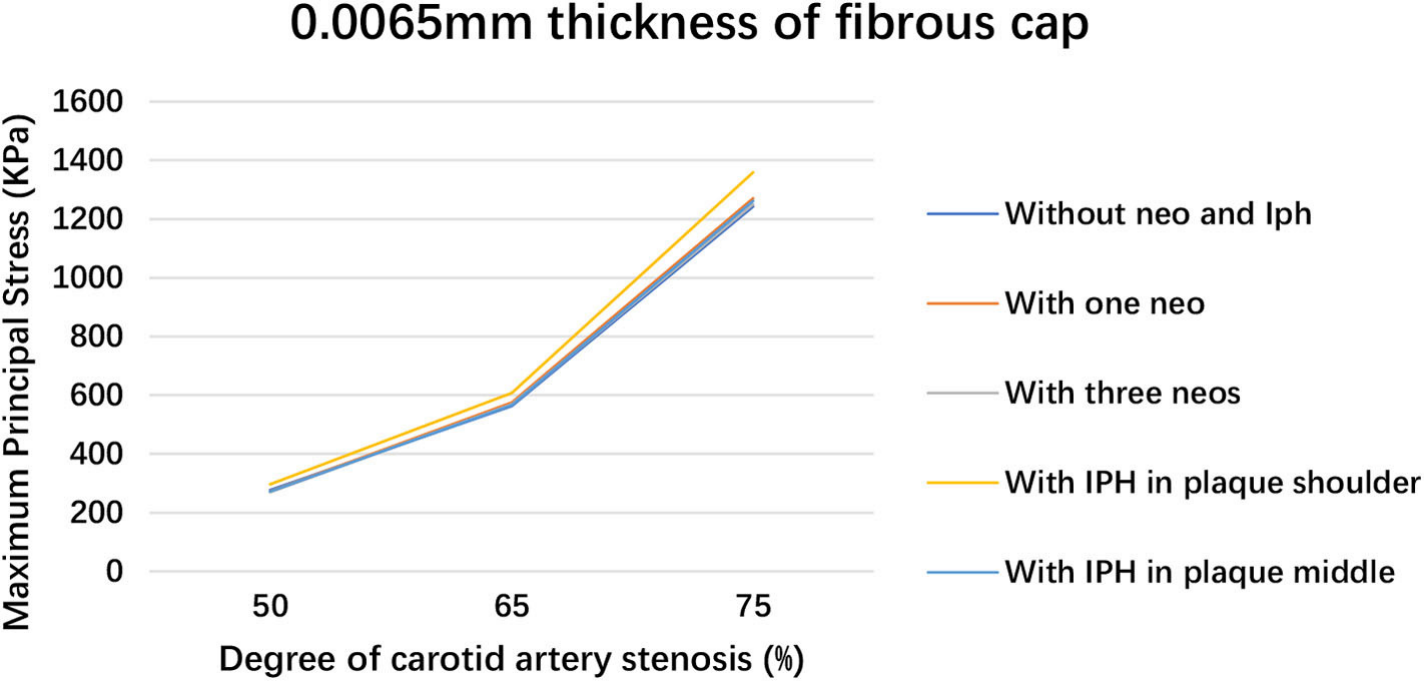
Plaque maximum principal stress within 0.0065 mm fibrous cap thickness when changing luminal stenosis.

**Figure 7:**
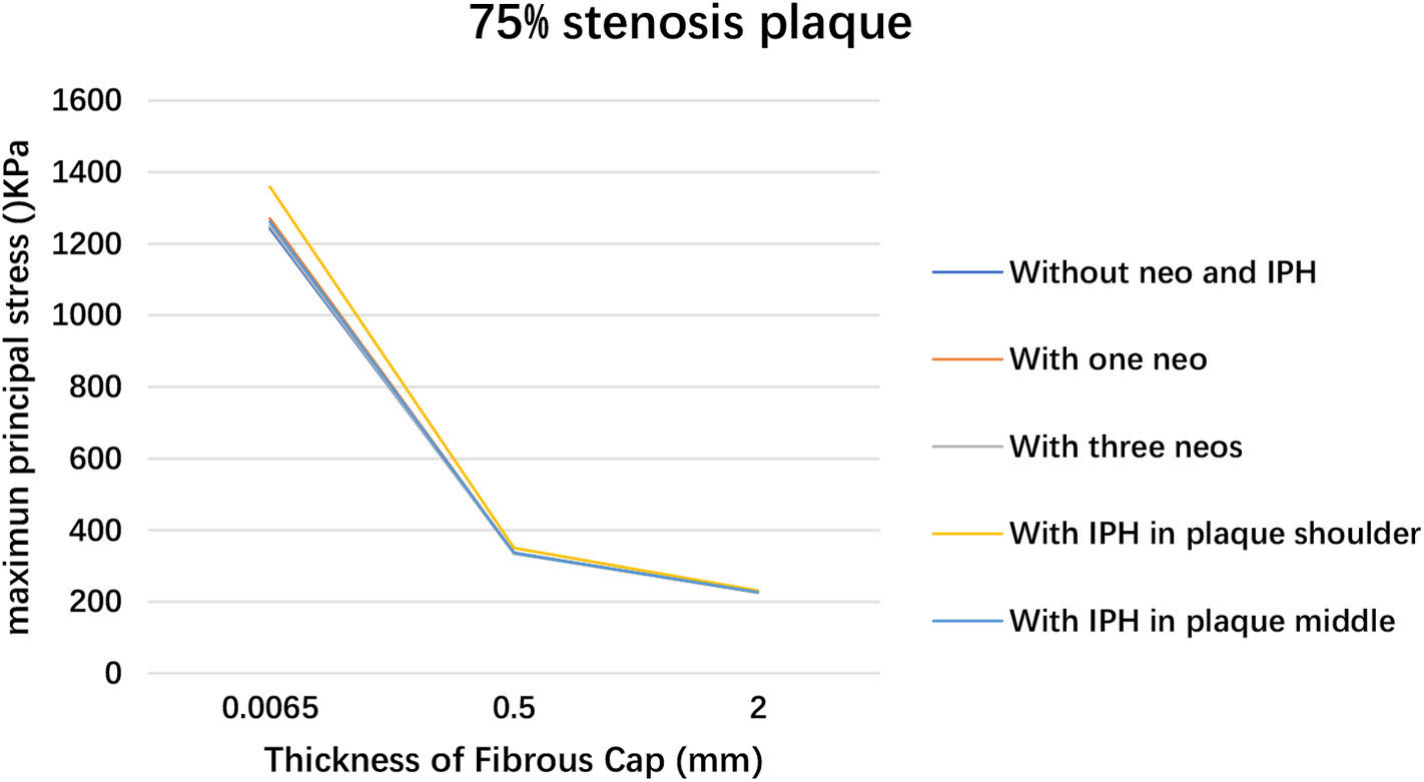
Plaque maximum principal stress variation within 75% stenosis luminal stenosis when changing fibrous cap thickness.

The deformation results of plaque with 65 and 75% luminal stenoses were illustrated in the Supplementary Figures 2–5. The maximum principal stress and strain in plaque with different degrees of luminal stenosis and fibrous cap thickness are summarized in Table 2. The maximum principal stresses are recorded in the thin fibrous cap geometry that it was 276.99, 563.67, and 1242.6 kPa for 50, 65, and 75% luminal stenosis, respectively. A higher degree of luminal stenosis was subjected to a higher maximum principal stress and strain in all the geometries with 0.0065 mm fibrous cap thickness (see Figure 6). And the maximum principal stress grows along with the thinning of the fibrous cap in the geometry with 75% luminal stenosis (see Figure 7). As shown in Table 2, the largest maximum principal stress was recorded in the geometries with 75% stenosis and the thickness of fibrous cap varied from 2 to 0.0065 mm. The maximum principal strain of plaque with 0.0065mm fibrous ca thickness increased significantly with the degree of the luminal stenosis. Consequently, The plaques observed, with (i) 65% stenosis, 0.0065 mm thickness, and (ii) 75% stenosis, 0.5 and 0.0065 mm thickness, were unstable and vulnerable. Moreover, for an artery with high degree of luminal stenosis and a plaque with thin fibrous cap thickness, a higher maximum principal stress was observed when neovascularization or IPH was presented compared to those without neovascularization or IPH in most cases. And, a relatively higher maximum principal stress was observed when IPH existed in plaque shoulder than in plaque middle.

## 4. Discussion

Vulnerable plaque in the carotid artery is a critical cause of many cardiovascular diseases like stroke and ischemic attacks (Schinkel et al., 2020). Neovascularization and IPH contribute significantly to the rupture of atherosclerotic plaques (Van der Veken et al., 2016; Demeure et al., 2017; Yang et al., 2020). Wang et al. (2020) found that increased carotid neovascularization was significantly related to aggravated cerebral white matter lesions in 269 participants based on CEUS. Camps-Renom et al. (2020) demonstrated that neovascularization was an independent predictor of ischemic stroke recurrence, based on 78 patients with carotid atherosclerosis. Literatures (Mark et al., 2020a,b; Yang et al., 2020) discovered that IPH had a close connection with a high risk of cardiovascular diseases. In addition, Teng et al. (2012) found that the computed parameters of stress and strain in a carotid artery plaque increased as the distance between the neovessel and the artery lumen decreased. There is a big potential for large deformation and high mechanical loading variation in an intraplaque neovessel. Huang et al. (2012) quantified the effect of IPH on plaque wall stress by using a 3D fluid–structure interaction model based on the magnetic resonance image; the authors found that the critical plaque wall stress and strain increased in geometries with IPH, relative to geometries without IPH. Additionally, fibrous cap thickness and luminal stenosis have also been demonstrated as two critical parameters that could induce plaque rupture (Li Z. Y. et al., 2006; Alegre-Marténez et al., 2019; Zareh et al., 2019). A plaque with thinner fibrous cap thickness and larger lipid core was more likely to rupture and cause stroke. Huang et al. (2012) have indicated that there was a connection between fibrous cap thickness and IPH occurrence based on the analysis by fluid–structure interaction. Therefore, to be consideration of fibrous cap thickness and luminal stenosis are necessary for analyzing the stress and strain effect of neovascularization and IPH of plaque.

In this paper, we have constructed an idealized geometry of the carotid artery with atherosclerotic plaque based on the CEUS image. The four factors of plaque rupture, namely neovascularization, IPH, fibrous cap thickness, and luminal stenosis, were evaluated. A one-way fluid–structure interaction model was employed to calculate the structural stress and strain in the carotid atherosclerotic plaque. The simulation results validated the conclusion mentioned above that the two factors of fibrous cap thickness and luminal stenosis degree play a significant role in the progression of carotid atherosclerotic plaque. The results of the one-way fluid–structure interaction model in this paper were consistent with the current literature (Li Z. Y. et al., 2006; Teng et al., 2012). Therefore, it is reasonable to assume the role of IPH and neovascularization distributions in the formation of carotid atherosclerotic plaque. IPH is one of the important factors of carotid atherosclerotic plaque, especially in vulnerable plaque (Saba et al., 2019b). It can accelerate the enlargement of the lipid core that can increase the risk of the plaque rupture.

In our work, two types of IPHs have been evaluated, and stress and strain were determined within the plaque were observed. It was a critical indication that IPH distributed at the shoulder of the plaque showed a higher risk to rupture compared to the plaque with IPH distributed in the middle area regardless of the fibrous cap thickness and the luminal stenosis degree (see Table 2). For the plaque with IPH in the plaque shoulder, 75% stenosis, and thin fibrous cap thicknesses, the maximum principal stress increased on an average to 34.47 kPa compared to the plaque with IPH in the plaque middle. In the plaque with 50 and 65% stenosis, the maximum principal stress increased on an average to 8.983 and 14.526 kPa, respectively. Besides, the plaque with IPH in the plaque shoulder had a 116.3 kPa higher maximum principal stress than the plaque without IPH, especially for the plaque with 75% stenosis and 0.0065 mm fibrous cap thickness. Therefore, the location of the plaque shoulder was a high-risk site for plaque rupture frequently. Additionally, IPH was not a negligible factor for assessing plaque vulnerability, and there was guidance by B-model ultrasonography to distinguish IPH (Gao et al., 2017; Zheng et al., 2020a,b).

Previous studies had (Huang et al., 2008; Hoogi et al., 2011; Van der Veken et al., 2016; Demeure et al., 2017) demonstrated that the feature of neovascularization also played an important role in the progression of plaque, and it may be associated with the possibility of stroke occurrence. Therefore, neovascularization within the plaque is important. The neovascularization can be visualized by CEUS imaging, and then help the cardiologist to observe the deformation of the neovascularization. However, the effect of the neovascularization on plaque risk only depended on the cardiologist's subjective diagnosis, according to the distribution and density of the IPH. Therefore, in this paper, the neovascularization was studied by modeling the neovessel geometry within the plaque. Figures 4, 5 show the deformation of the neovessel in 50% luminal stenosis in conjunction with different fibrous cap thickness. The larger deformation of the neovessel was found with a thinner fibrous cap. The deformation of the neovessel was in line with the discovery carotid artery plaque in CEUS images. Table 2 indicates that the maximum principal stress and strain was similar between geometries with neovessel and without neovessel as a result of the similar distribution and density of the neovessels. However, Figures 4, 5 and Supplementary Figures 1–5 show the occurrence of neovessel deformation. That is to say, the neovessel underwent the loading of mechanical stress and had the potential to rupture and then lead to IPH.

Additionally, according to experimental results of fluid flow, the distribution of plaque stress and strain mainly focused on the proximal surface of the plaque, regardless of luminal stenosis and fibrous cap thickness. Choi et al. (2015) have found that axial plaque stress and wall shear stress in the upstream were higher than in downstream. These findings demonstrated that the site of plaque shoulder was more likely to tend to rupture because it is undergoing shear stress induced by the blood flow (Malek, 1999). The variation of shear stress could alter the characteristics of the endothelial cell, including its morphology, function, proliferation, and even vascular injury (Malek, 1999; Chatzizisis et al., 2009). Therefore, the stress and strain distribution in the proximal surface of plaque fluid flow has an important role in adjudging plaque rupture. Additionally, the neovessel was only made up of little endothelial cells and the vulnerability of neovessel was poor. Therefore, it is of significance to improve the assessment of vulnerable plaque by analyzing the effect of hemodynamic stress and strain on the plaque with neovascularization and IPH.

There are some limitations to this study. First, it was a theoretical research, and the geometry of the carotid artery was idealized. The carotid bifurcation was not taken into account, as the neovessel and the IPH were the focus of this paper. Second, only two types of neovessels were analyzed. The shape in conjunction with the location of the neovessel was flexible and variable. McCarthy et al. (1999) found that the neovessel within a plaque may exist in the medial, the lateral corners, and the bottom of the plaque. Third, the material properties of the neovessel were assumed from literatures, and this may have resulted in the parameters of maximum principal stress and strain levels being below or over the actual value. There is a small difference in the value of the maximum principal stress between the plaque with neovessel and without neovessel. The material properties, shape, and location of the neovessel may be the most likely reason for it. Another limitation is that this idealized geometry was two-dimensional. Previous studies have illustrated the histology image of the transversal slice of the plaque, indicating the distribution of microvasculature could either axial or transversal (Lu et al., 2015; Guo et al., 2018, 2019). These neovessels were with significant variation of the size of the neovessels. While previous studies focus on the axially distributed neovessel, our results suggested that the mechanical effects of transversal distributed neovessel was also important to the vulnerability of the plaque. Also, in comparison to the previous study (Teng et al., 2015), the morphology of IPH used in the present study was generally consistent with it, even if the CEUS imaging is less applicable for detecting IPH. However, as the neovessel was more complex with branches pointing toward different directions and dimensions, further study was required to establish a more realistic geometry of the plaque with 3-dimensional distribution of the microenvironment for a more comprehensive understanding of the mechanics property of the plaque.

## 5. Conclusion

In conclusion, a numerical analysis of the stress and strain within carotid artery plaque was conducted. We verified that the plaque with thin fibrous cap and serious luminal stenosis has a high risk of rupture. At the same time, neovascularization and IPH were also studied. Pulsatile blood flow was related to the deformation phenomenon of neovessel occurrence in intraplaque observed in CEUS image. Also, the IPH has the potential to increase the possibility of plaque rupture. Therefore, IPH and neovascularization may also be considered to be the features for plaque progression and even rupture, besides the two key factors of fibrous cap thickness and the degree of luminal stenosis.

## Data Availability Statement

The original contributions presented in the study are included in the article/Supplementary Material, further inquiries can be directed to the corresponding author/s.

## Author Contributions

ZL and XW: conceptualization. YW: methodology, validation, investigation, and writing-original draft. XL: supervision, project administration, and writing - review & editing. SL, ZL, XL, and LR: funding acquisition. SH, YH, and LR: data curation.

## Conflict of Interest

The authors declare that the research was conducted in the absence of any commercial or financial relationships that could be construed as a potential conflict of interest.
