# Supplementary material for: Studying the Factors of Human Carotid Atherosclerotic Plaque Rupture, by Calculating Stress/Strain in the Plaque, Based on CEUS Images: A Numerical Study
Source: Front Neuroinform. 2020 Nov 24;14:596340. doi: 10.3389/fninf.2020.596340 (PMC7721669; doi:10.3389/fninf.2020.596340)
Supplement: Supplementary file 1 [file Data_Sheet_1.PDF]

Supplementary Material

1 SUPPLEMENTARY DATA

1.1 Figures

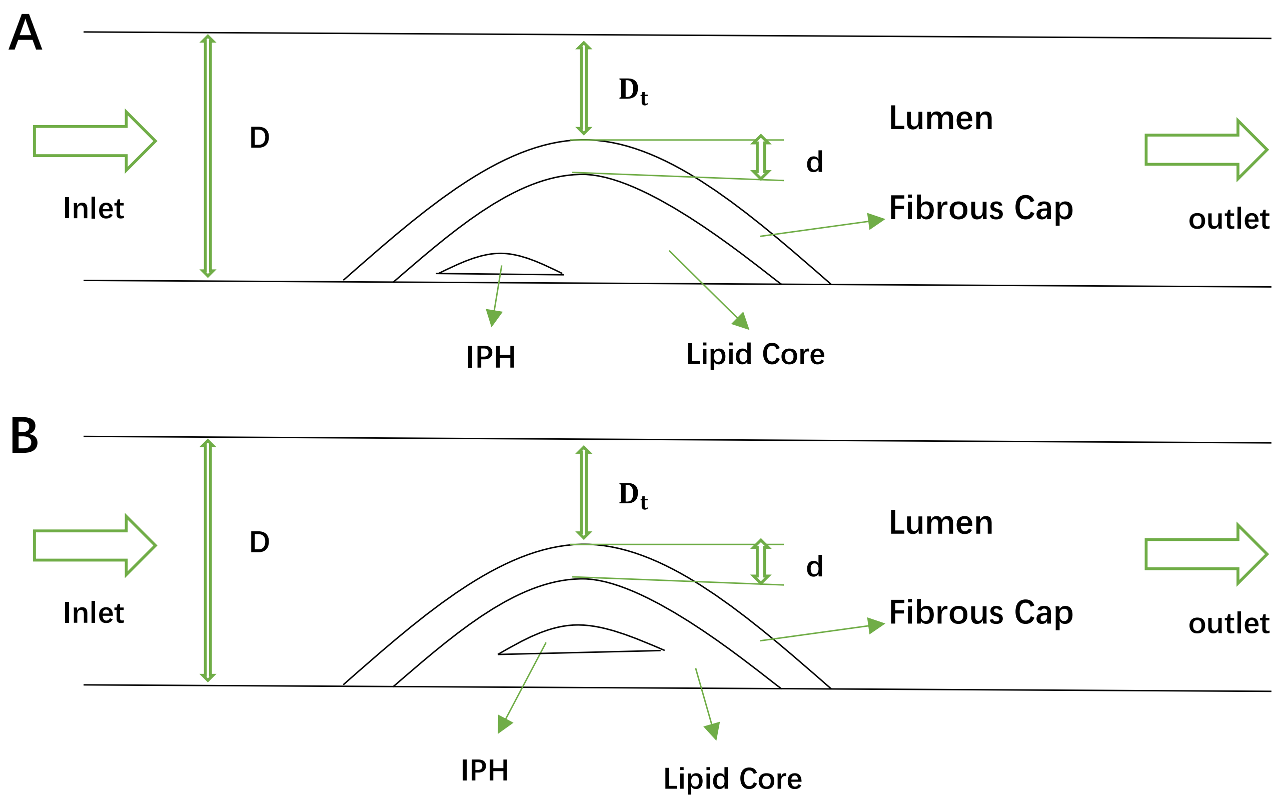

Figure S1. The 2D geometries of the ideal carotid artery with plaque and IPH, (A) is the geometry with IPH in plaque shoulder, (B) is the geometry with IPH in plaque middle.

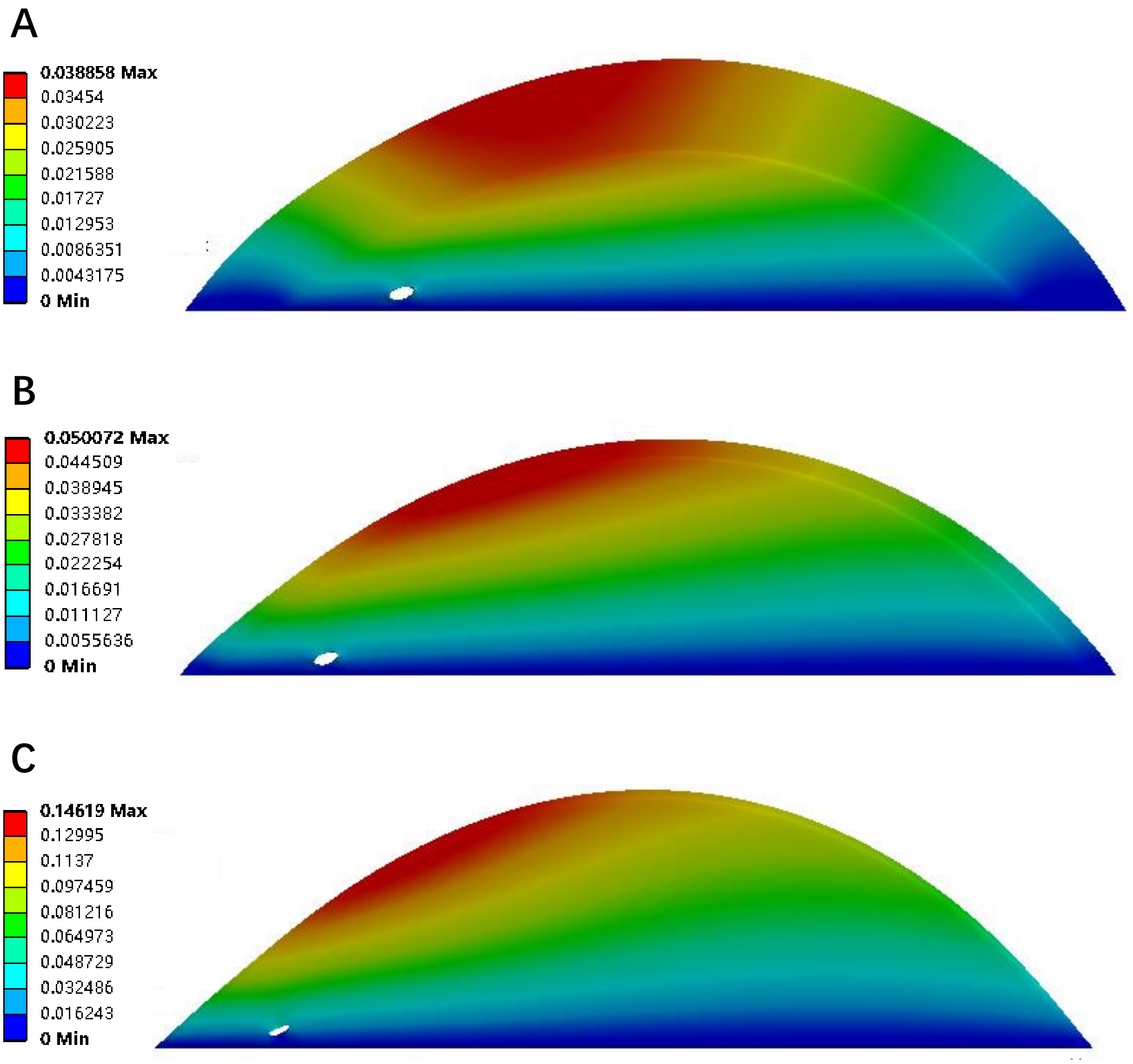

Figure S2. Deformation results of plaque with 65% luminal stenosis, one neovessel, and different thickness of fibrous cap. The fibrous cap thicknesses of subfigures (A), (B), and (C) were 2mm, 0.5mm, 0.0065mm, respectively.

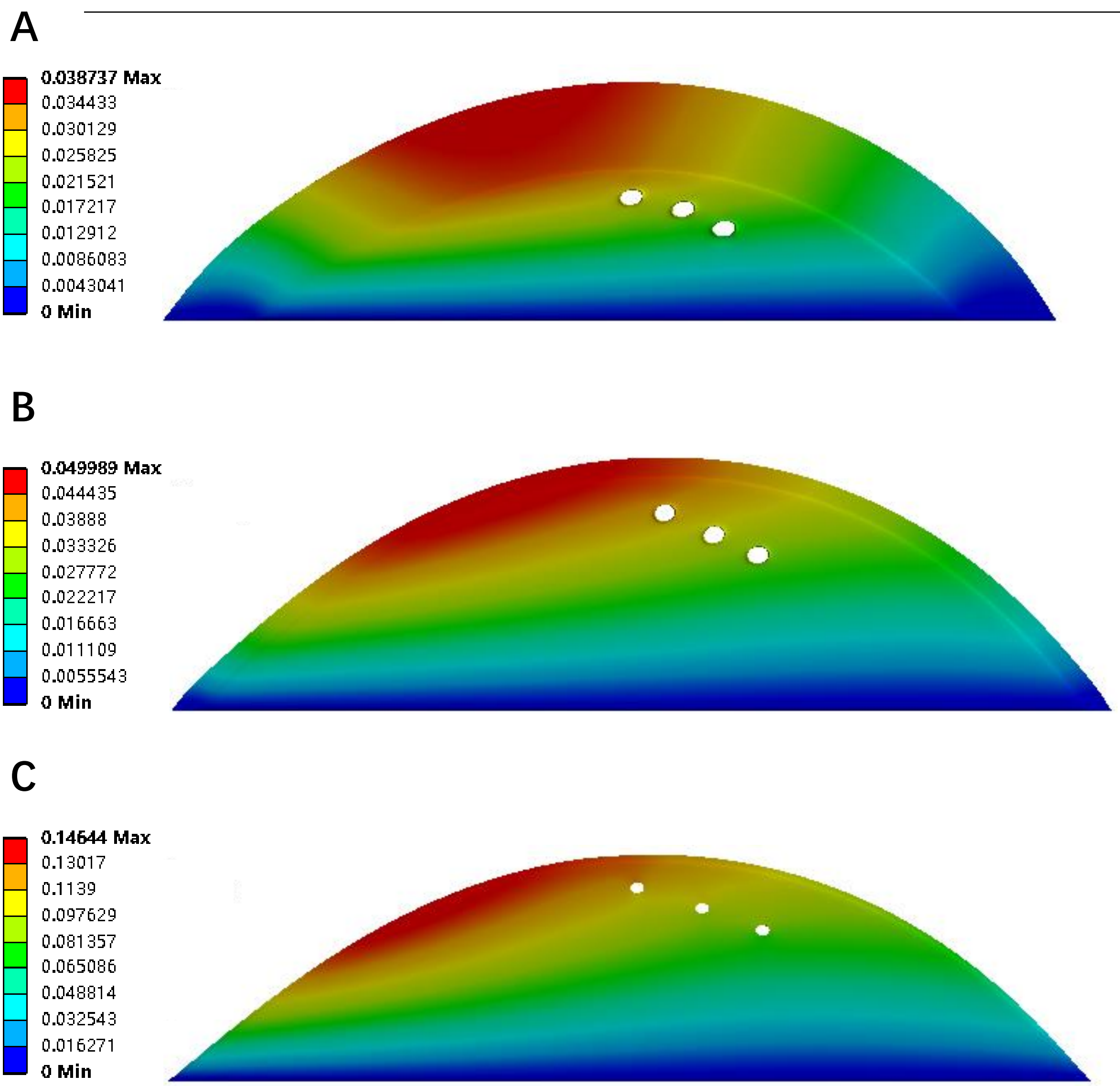

Figure S3. Deformation results of plaque with 65% luminal stenosis, one neovessel, and different thickness of fibrous cap. The fibrous cap thicknesses of subfigures (A), (B), and (C) were 2mm, 0.5mm, 0.0065mm, respectively.

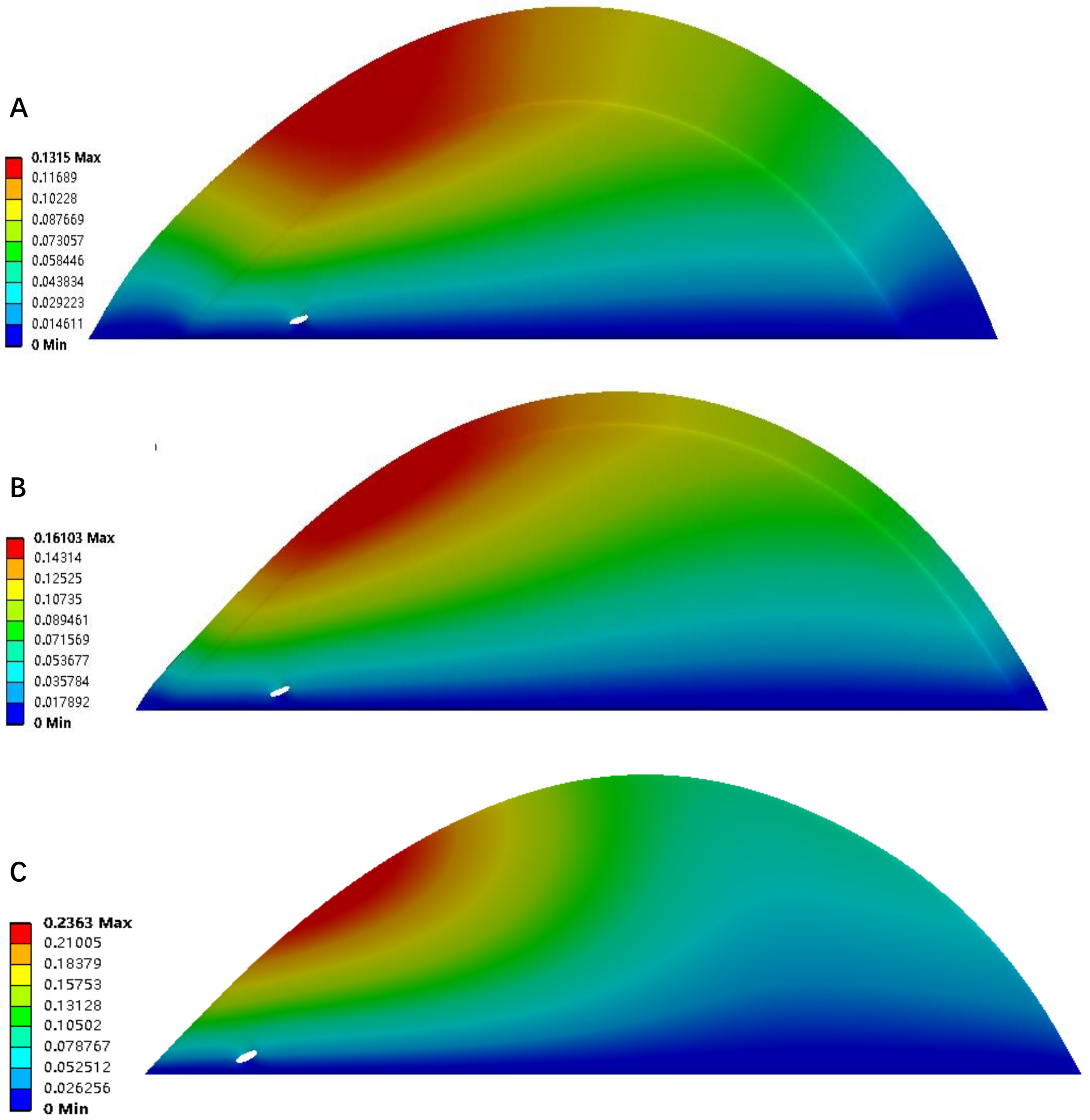

Figure S4. Deformation results of plaque with 75% luminal stenosis, one neovessel, and different thickness of fibrous cap. The fibrous cap thicknesses of subfigures (A), (B), and (C) were 2mm, 0.5mm, 0.0065mm, respectively.

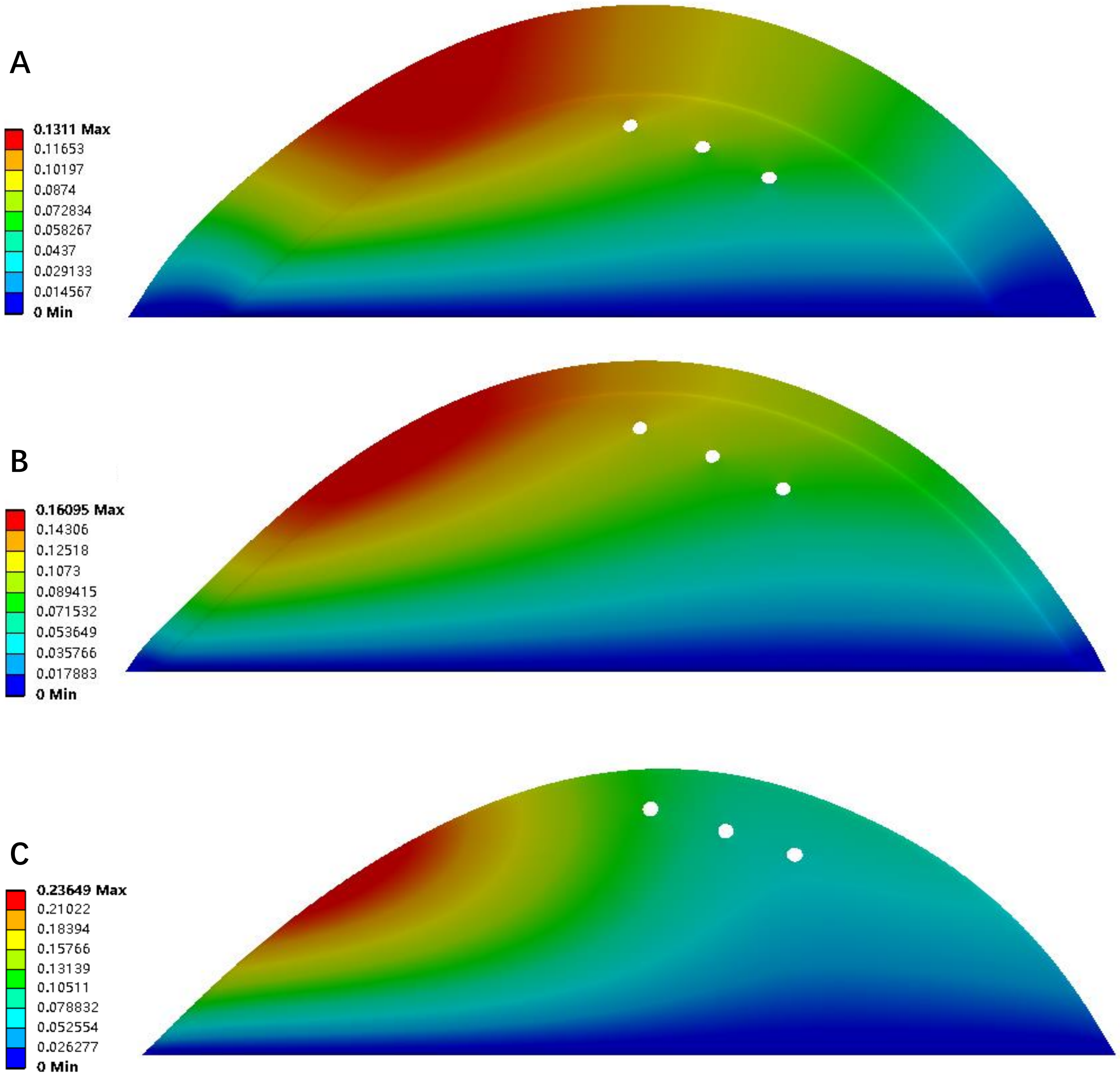

Figure S5. Deformation results of plaque with 75% luminal stenosis, three neovessels, and different thickness of fibrous cap. The fibrous cap thicknesses of subfigures (A), (B), and (C) were 2mm, 0.5mm, 0.0065mm, respectively.
